# Nonreciprocal Magnetic Coupling Using Nonlinear Meta‐Atoms

**DOI:** 10.1002/advs.202001443

**Published:** 2020-07-23

**Authors:** Xiaoguang Zhao, Ke Wu, Chunxu Chen, Thomas G. Bifano, Stephan W. Anderson, Xin Zhang

**Affiliations:** ^1^ Department of Mechanical Engineering Boston University Boston MA 02215 USA; ^2^ Department of Radiology Boston University Medical Campus Boston MA 02118 USA; ^3^ Photonics Center Boston University Boston MA 02215 USA

**Keywords:** coupled mode theory, magnetic coupling, meta‐atoms, nonlinear, nonreciprocity

## Abstract

Breaking Lorentz reciprocity is fundamental to an array of functional radiofrequency (RF) and optical devices, such as isolators and circulators. The application of external excitation, such as magnetic fields and spatial–temporal modulation, has been employed to achieve nonreciprocal responses. Alternatively, nonlinear effects may also be employed to break reciprocity in a completely passive fashion. Herein, a coupled system comprised of linear and nonlinear meta‐atoms that achieves nonreciprocity based on the coupling and frequency detuning of its constituent meta‐atoms is presented. An analytical model is developed based on the coupled mode theory (CMT) in order to design and optimize the nonreciprocal meta‐atoms in this coupled system. Experimental demonstration of an RF isolator is performed, and the contrast between forward and backward propagation approximates 20 dB. Importantly, the use of the CMT model developed herein enables a generalizable capacity to predict the limitations of nonlinearity‐based nonreciprocity, thereby facilitating the development of novel approaches to breaking Lorentz reciprocity. The CMT model and implementation scheme presented in this work may be deployed in a wide range of applications, including integrated photonic circuits, optical metamaterials, and metasurfaces, among others.

## Graphical abstract

Breaking Lorentz reciprocity is the foundation of an array of functional electromagnetic devices, and often requires external excitation. Magnetically coupled linear and nonlinear meta‐atoms are experimentally employed to achieve giant and controllable nonreciprocity. The physics of such a passive and magnet‐free nonreciprocal isolator are explored by using coupled mode theory for generalization of the presented approach.

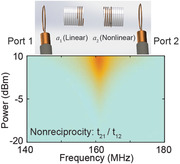

As a fundamental principle in electromagnetics and optics, reciprocity is valid in most linear and time‐invariant materials that possess symmetric permittivity and permeability tensors.^[^ ^1^ ^]^ In certain applications; however; it is desirable to break reciprocity as nonreciprocity is the foundation of an array of functional devices, including isolators, circulators, and directional amplifiers.^[^ ^2^ ^]^ A conventional approach to achieving nonreciprocity in electromagnetics is to employ the gyromagnetic effect, or Faraday rotation, in magnetized ferrimagnetic materials. In such devices, the external magnetic field induces asymmetric permittivity and permeability tensors, which lead to transmission coefficients dependent on the wave propagation direction.^[^ ^3^ ^]^ However, the relatively low strength of magneto‐optical effects makes it difficult to create compact, high‐efficiency nonreciprocal devices using such conventional magneto‐optical effects.^[^ ^4^ ^]^ Furthermore, it is technically challenging to integrate gyromagnetic materials and magnets on chip. Therefore, the realization of nonreciprocity in a magnet‐free fashion is of fundamental importance.

Early efforts to implement magnet‐free, nonreciprocal transmission line devices using sequentially actuated switches have been reported.^[^ ^5^, ^6^ ^]^ Recently, with the development of high‐speed electronic and photonic devices and the increasing demands of integrated on‐chip, nonreciprocal devices, spatial–temporal modulation in the constituent materials in electromagnetic resonators,^[^ ^7^, ^8^, ^9^, ^10^, ^11^ ^]^ optical metamaterials,^[^ ^12^, ^13^, ^14^ ^]^ and integrated photonic circuits,^[^ ^15^, ^16^, ^17^ ^]^ among others,^[^ ^18^ ^]^ has been employed to break the time‐reversal symmetry by breaking time‐invariance. Generally speaking, the temporally and spatially variant electromagnetic properties in these devices act as a synthetic linear momentum to the structures that breaks the time‐reversal symmetry in travelling waves. The realization of nonreciprocity based on spatial–temporal modulation requires the local variance of these properties at frequencies comparable to the carrier wave frequencies. In addition, the phase of the electromagnetic property modulation should be matched in order to achieve the desired nonreciprocal response. The stringent requirements on the modulation frequency and phase matching increase the complexity and ultimately bound the frequency bands of such devices.

An alternative route toward achieving magnet‐free nonreciprocity is to leverage nonlinearity.^[^ ^1^ ^]^ Metamaterials and metasurfaces exhibit strong nonlinear effects if quantum materials are employed in their construction, yielding a powerful platform by which to achieve high harmonic generation,^[^ ^19^ ^]^ wave mixing,^[^ ^20^ ^]^ field induced electron emission,^[^ ^21^ ^]^ holographic imaging,^[^ ^22^ ^]^ and saturable absorption,^[^ ^23^ ^]^ among other properties. Nonlinear metamaterials are an effective approach toward achieving nonreciprocal optical devices in the infrared and visible regimes.^[^ ^24^, ^25^, ^26^ ^]^ In nonreciprocal systems based on nonlinear materials, the constituent structures are spatially asymmetric and the field intensity within the nonlinear materials varies as a function of incident direction and, thereby, the transmission coefficient depends on the propagation direction.^[^ ^27^ ^]^ For instance, cascaded meta‐atoms have been developed to achieve one‐way propagation of radio‐frequency (RF) signals.^[^ ^28^ ^]^ In order to extend the frequency of nonlinear nonreciprocal devices, serially connected nonlinear Fano and Lorentz resonators have been exploited to achieve high isolation ratios.^[^ ^29^ ^]^ Nonreciprocity in optical regimes, nonlinear Faraday rotation, and nonlinear topological states have been achieved in this fashion.^[^ ^30^, ^31^, ^32^ ^]^ In addition, metamaterials capable of enhancing local magnetic fields have been developed by tailoring metamaterial designs for various applications, including ultra‐sensitive biological sensing.^[^ ^33^, ^34^, ^35^, ^36^, ^37^ ^]^ In this article, we present a paradigm by which to achieve nonreciprocal RF transmission via coupled linear and nonlinear resonating magnetic meta‐atoms. A lumped‐parameter, analytical model based on the coupled mode theory (CMT) is developed to theoretically study the nonreciprocal response. Experimental results demonstrate a marked degree of contrast between forward and backward transmission at the resonance frequencies. Our study elucidates the fact that the coupling between meta‐atoms affects the degree of nonreciprocal isolation contrast. Trade‐offs between the forward transmission coefficient and the isolation contrast need to be considered during the design of nonreciprocal systems. Notably, the concise theoretical model developed herein to predict the response of our specific coupled system is readily generalizable and may be considered a new paradigm in nonlinear, nonreciprocal device design.

In theoretically modeling a coupled, nonreciprocal system, we first consider a system consisting of coupled linear and nonlinear resonators, as shown in **Figure** **1**a, the structure of which is inherently asymmetric. In such a coupled system, we may employ the CMT to calculate the mode amplitudes *a*_1_ and *a*_2_ of the resonators:^[^ ^38^, ^39^, ^40^ ^]^

(1) $$\frac{da_{1}}{dt}=\left[j\omega_{1}-\frac{1}{\tau_{e1}}-\frac{1}{\tau_{o1}}\right]a_{1}+jka_{2}+\sqrt{\frac{2}{\tau_{e1}}}s_{1+}$$

(2) $$\frac{da_{2}}{dt}=\left[j\omega_{2}-\frac{1}{\tau_{e2}}-\frac{1}{\tau_{o2}}\right]a_{2}+jka_{1}+\sqrt{\frac{2}{\tau_{e2}}}s_{2+}$$

in which *ω_m_* is the resonant frequency of resonator *m* (*m* = 1 or 2 is the resonator number), 1/*τ*_*e*m_ and 1/*τ*_*o*m_ are, respectively, the decay rates due to emission and ohmic loss, *k* is the coupling coefficient between the two resonators, and *s*_1+_ and *s*_2+_ are the input signals from port 1 and port 2. By converting the equations to the frequency domain, we obtain

(3) $$j\omega \left[\begin{array}{c} a_{1}\\ a_{2} \end{array}\right]=j\left[\begin{array}{cc} \omega_{1}+j\gamma_{1} & k\\ k & \omega_{2}+j\gamma_{2} \end{array}\right]\left[\begin{array}{c} a_{1}\\ a_{2} \end{array}\right]+\left[\begin{array}{cc} k_{1} & 0\\ 0 & k_{2} \end{array}\right]\left[\begin{array}{c} s_{1+}\\ s_{2+} \end{array}\right]$$

in which *γ*_1_= (1/*τ*_*e*1_ + 1/*τ*_*o*1_) and *γ*_2_= (1/*τ*_*e*2_ + 1/*τ*_*o*2_) are the damping rates of resonators 1 and 2, respectively, and *k*_1_ =$\sqrt{2/\tau_{e1}}$ and *k*_2_ = $\sqrt{2/\tau_{e2}}$ are the coupling coefficients of resonator 1 to port 1 and resonator 2 to port 2, respectively. Resonator 1 is linear and, therefore, the resonance frequency *ω*_1_ = *ω_o_*_1_is fixed. Resonator 2 is nonlinear, with its resonance frequency (*ω*_2_) related to the mode amplitude *a*_2_ by *ω*_2_ = *ω*_*o*2_(1 − *λ*_0_|*a*_2_|), where *ω*_*o*2_ is the original resonance frequency and *λ*_0_ is the nonlinearity coefficient.^[^ ^40^ ^]^ By solving Equation (1), we derive the mode amplitude of the resonators and, thereby, calculate the output signals *s*_1−_ and *s*_2−_ at ports 1 and 2 by $s_{1-}=\sqrt{2/\tau_{e1}}a_{1}$ and $s_{2-}=\sqrt{2/\tau_{e2}}a_{2}$. The transfer matrix of the system can be calculated by:

(4) $$\left[\begin{array}{c} s_{1-}\\ s_{2-} \end{array}\right]=\left[\begin{array}{cc} r_{11} & t_{12}\\ t_{21} & r_{22} \end{array}\right]\left[\begin{array}{c} s_{1+}\\ s_{2+} \end{array}\right]$$

where *r*_11_ and *r*_22_ are the reflection coefficients at ports 1 and 2, respectively, and *t*_21_ and *t*_12_ are the forward (from port 1 to port 2) and backward (from port 2 to port 1) transmission coefficients, respectively.

**Figure 1:**
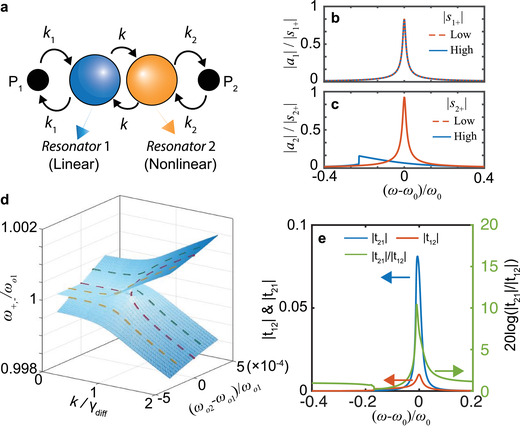
Working principle of the nonreciprocal system based on coupled linear‐nonlinear resonators. a) Schematic of the coupled system, in which resonator 1 is linear while resonator 2 is nonlinear. b,c) Mode amplitude of the linear resonator (b) and the nonlinear resonator (c). d) Riemann surface of the real part of the eigenvalues for different coupling factors and degrees of frequency detuning. e) The forward (*t*_21_) and backward (*t*_12_) transmission coefficients and isolation contrast as a function of frequency for the nonreciprocal system under high excitation power.

Next, we analyze the resonance response of the uncoupled, standalone meta‐atom resonators by assuming *k* = 0 in Equation (3). Resonator 1 is linear and its resonance amplitude does not vary as a function of excitation strength, as shown in Figure 1b. In contradistinction, the nonlinear resonator (resonator 2) exhibits a strong excitation‐dependent resonance mode amplitude.^[^ ^40^ ^]^ During low‐power excitation, there is a strong resonance at the resonant frequency in the case of the nonlinear resonator, similar to the linear resonator. However, during high‐power excitation, a nonlinear, bi‐stable response emerges and the resonance amplitude of the nonlinear resonator is weak at its original resonance frequency, as shown in Figure 1c. Therefore, resonator 2 is functionally “off” during high‐power excitation and remains “on” during low‐power excitation, thereby providing a route toward nonreciprocity.

In the case of coupled meta‐atom resonators (*k* > 0), the system may be considered as a passive, parity‐time (PT) symmetric system when *γ*_1_ ≠ *γ*_2_.^[^ ^41^, ^42^ ^]^ The requisite difference in damping rates [defined as *γ*_diff_ = (*γ*_1_ − *γ*_2_)/2] between the resonators may be achieved by altering their geometries. First, we assume that both resonators are in the linear regime (low‐power excitation) and calculate the eigenvalues of the coupled system. The evolution of the real part of the eigenvalues, that is, the eigenfrequencies of the system, in two‐dimensional parameter space (*k*/*γ*_diff_ and Δ*ω* = (*ω_o_*_1_ − *ω*_*o*2_)/*ω_o_*_1_) is shown in Figure 1d. When the frequencies of the two resonators are matched (Δ*ω* = 0), the eigenfrequencies are split when the coupling strength is high (*k* >*γ*_diff_) and degenerate when the coupling strength is low (*k ≤ γ*_diff_). The special point (Δ*ω* = 0, *k = γ*_diff_ ) is the so‐called exceptional point for this passive PT‐symmetry system,^[^ ^43^ ^]^ at which both of the eigenvalues and eigenvectors of the coupled system are degenerated. We consider a system with low coupling strength in order to ensure a high degree of contrast between forward and backward transmission coefficients. As shown in Figure 1e, the transmission coefficients exhibit stark differences depending on the incident direction. The transmission coefficient from port 1 to port 2 (*t*_21_) is approximately tenfold larger than the transmission coefficient from port 2 to port 1 (*t*_12_). Ultimately, the nonreciprocal response of the coupled system stems from the nonlinear response in resonator 2.

In order to experimentally demonstrate the nonreciprocity in the coupled linear‐nonlinear resonant meta‐atom system, we measured the transmission coefficient between the inductively coupled resonators, as shown in **Figure** **2**a. The linear resonator was a metallic, helical meta‐atom, while the nonlinear resonator was a metallic, helical meta‐atom closely packed with a split‐ring resonator loaded by a varactor. The nonlinear meta‐atom exhibited an excitation‐dependent resonant response. The resonance frequency of the nonlinear meta‐atom (*ω*_2_) follows the theoretical model in Equation (3). Due to the voltage‐dependent capacitance of the varactor diode induced by the reversely‐biased P‐N junction, the capacitance is altered as the incident RF power changes. The nonlinear resonator may be considered as an inductor–capacitor (*LC*) resonator, thereby, its resonance frequency (*ω*_2_ ∝ 1/√*C*, where *C* is the varactor capacitance) is linearly dependent upon the mode amplitude and, thusly, exhibits a nonlinear optical response. The coupling coefficient was tunable by controlling the separation distance (*d*) between the resonators, and the resonance frequency detuning (Δ*ω*) was controlled by the geometry of the linear resonator (resonator 1). When Δ*ω* was equal to zero and the coupling factor was weak, asymmetric transmission (Figure 2b) was achieved with an incident power of 4 dBm. At the resonant frequency, *t*_21_ was ≈0.12 while *t*_12_ was ≈0.019, yielding ≈16.13 dB contrast. In the theoretical modeling, the experimental results may be fit by solving Equation (3) with an excitation amplitude |*s*_1,2+_| = 0.03 and a coupling factor *k* = 0.0018, as shown in Figure 2c. As discussed above, the transmission coefficient *t*_21_ is higher than *t*_12_ at the resonance frequency due to the nonlinearity in resonator 2 during high‐power excitation.

**Figure 2:**
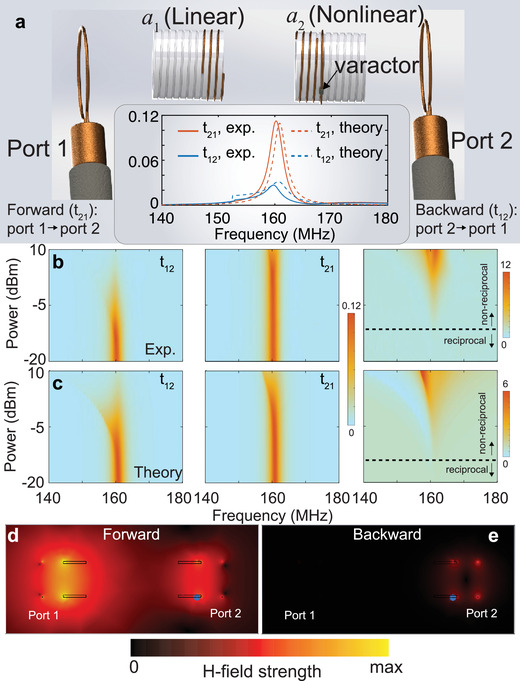
Experimental validation and analysis of the nonreciprocal response using the magnetically coupled meta‐atoms. a) Illustration of the experimental setup to characterize the nonreciprocal response. Inset: measured and analytically calculated forward (*t*_21_) and backward (*t*_12_) transmission coefficients when the incident RF power is 0 dBm. b) Measured and c) calculated forward (*t*_12_) and backward (*t*_21_) transmission and isolation contrast for varied incident RF power. (d) and (e) are the numerically simulated magnetic field distribution for forward and backward propagation, respectively.

Importantly, the nonreciprocity of the system is dependent upon the input power. We experimentally measured the transmission coefficients (*t*_21_ and *t*_12_) for varied incident RF power from −20 dBm to 10 dBm. As shown in Figure 2b, the transmission coefficients *t*_21_ and *t*_12_ at 160 MHz are both high in the case of low excitation power. We define the isolation contrast (IC) as the ratio between *t*_21_ and *t*_12_, that is, IC = 20log_10_|*t*_21_|/|*t*_12_|. When the excitation power was less than −10 dBm, the IC was lower than 3 dB, indicating that the response was reciprocal. When the excitation was higher than −10 dBm, the IC gradually increased and reached a peak value (≈18.3 dB) at an excitation power of 10 dBm. It should be mentioned that *t*_21_ begins to decrease when excitation power exceeds 6 dBm, which is a sign of the emergence of a nonlinear response in the case of forward propagation. By employing the CMT model, we obtained a similar incident power‐dependent response, as shown in Figure 2c. The agreement between the experimental results and theoretical calculation serves to validate the analytical model, providing insight into the physics of the nonreciprocal response.

Next, we studied the nonreciprocal response of the coupled meta‐atom system by utilizing numerical simulations with CST Studio Suite 2018. Since it is challenging to model the field‐dependent and dynamic response in the varactor, we consider the varactor as a static capacitance in the numerical model. In the case of forward propagation, the capacitance remains in its original value and resonator 1 and 2 are both in resonant states (Figure 2d), yielding a high transmission coefficient. In the case of backward propagation, the capacitance is altered to a larger value due to the nonlinear effect, such that resonator 2 is in a non‐resonant state, yielding a low transmission coefficient (Figure 2e). Ultimately, the nonreciprocal transmission coefficient is due to the field‐dependent capacitance that yields an alteration in the resonant state of resonator 2.

Subsequently, we experimentally measured the transmission coefficient as a function of the design parameters of the helical resonators. We modified the resonance frequency difference by changing the resonance frequency of *a*_1_, that is, *ω_o_*_1_, and modified the coupling factor between *a*_1_ and *a*_2_ by changing the distance between the resonators. In one experiment, we increased the frequency of the linear meta‐atom and modified the distance between meta‐atoms to obtain the results shown in **Figure** **3**. In the forward transmission spectrum, there were two resonance modes, of which the lower‐frequency mode (*f*_2_) was dominated by the resonance of the nonlinear meta‐atom *a*_2_, while the higher‐frequency mode (*f*_1_) was dominated by the linear meta‐atom *a*_1_. The forward transmission coefficient was nearly independent of the incident power, as shown in Figure 3a, which differs from the frequency‐matched condition (Figure 2b). Backward transmission was highly dependent on the incident power, and the transmission at *f*_2_ decreased as the incident power increased, as shown in Figure 3b. The maximum isolation contrast was approximately 19.9 dB at 162 MHz when the input power was 10 dBm, as shown in Figure 3c. Using our analytical model within the framework of the CMT, we may also readily calculate the response for frequency‐mismatched cases. As shown in Figure 3d–f, we obtained the calculated forward and backward transmission coefficients for Δ*ω* = 0.045 and *k* = 0.0031, which agree well with the measured results. The theoretical maximum isolation contrast is 20 dB for the highest degree of excitation power.

**Figure 3:**
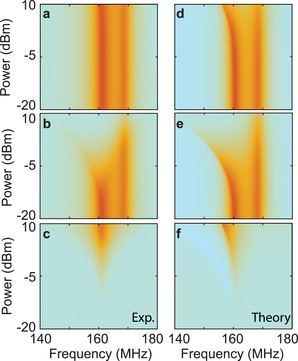
a–c) Experimentally measured forward transmission coefficient (a), backward transmission coefficient (b), and isolation contrast (c) for different incident power in the frequency‐detuned case. d–f) Corresponding theoretically calculated forward transmission coefficient (d), backward transmission coefficient (e), and isolation contrast (f).

The ideal nonreciprocal response is such that forward transmission is independent of variations in power and the backward transmission coefficient approaches 0 in the case of high incident power. The power‐dependence of the transmission coefficients (*t*_12_ and *t*_21_) and the isolation contrast of the coupled meta‐atom system are shown in **Figure** **4**a,b. When Δ*ω* = 0 and *k* = 0.001, the maximum forward transmission amplitude begins to decrease when the incident power exceeds 4 dBm. When Δ*ω* = 0.045 and *k* = 0.0031, the forward transmission amplitude at the resonance frequency is stable and approximates 0.067 for the incident power up to 10 dBm, while the maximum backward transmission amplitude decreases to 0.0068 at the highest input power, which approaches the aforementioned ideal nonreciprocal response. However, when Δ*ω* = 0 and *k* = 0.001, the undesired resonant frequency forward transmission amplitude decrease occurs when the incident power exceeds 5 dBm. The decrease in the resonant frequency backward transmission amplitude is larger than the decrease in forward transmission amplitude, consistent with a nonreciprocal response. The calculated forward and backward transmission amplitudes, as a function of resonant frequency, are also plotted in Figure 4a, which quantitatively agree with the measured results.

**Figure 4:**
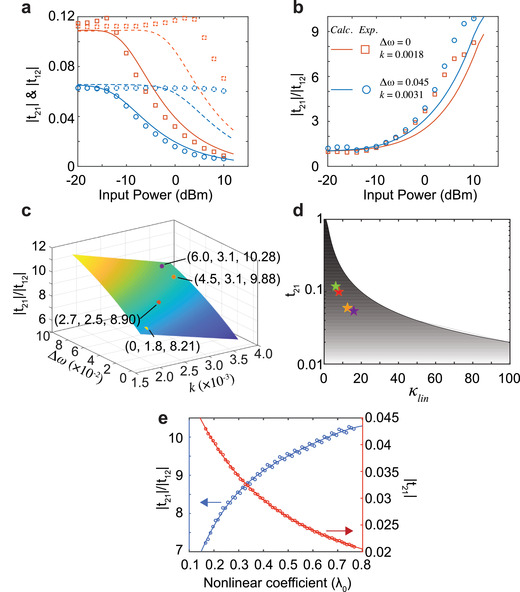
The bounded transmission coefficient in the nonreciprocal response. a) Experimental and calculated transmission coefficients at the resonance frequency of the nonlinear meta‐atoms for varied input power. red solid line: calculated *t*_12_ for Δ*ω* = 0 and *k* = 0.001; red dashed line: calculated *t*_21_ for Δ*ω* = 0 and *k* = 0.001; red solid squares: measured *t*_12_ for Δ*ω* = 0 and *k* = 0.001; red dashed squares: measured *t*_21_ for Δ*ω* = 0 and *k* = 0.001; blue solid line: calculated *t*_12_ for Δ*ω* = 0.047 and *k* = 0.0031; blue dashed line: calculated *t*_21_ for Δ*ω* = 0.047 and *k* = 0.0031; blue solid circles: measured *t*_12_ for Δ*ω* = 0.047 and *k* = 0.0031; blue dashed circles: measured *t*_21_ for Δ*ω* = 0.047 and *k* = 0.0031. b) Isolation contrast versus input power for different configurations of meta‐atoms. c) Calculated isolation contrast for different Δ*ω* and *k* for the coupled, nonreciprocal system and experimental results (dots). d) Theoretical bound of forward transmission versus asymmetric ratio (*κ*_lin_) of the coupled system and experimental results (stars) for four different configurations. The shaded area is the allowable transmission coefficient for varied *κ*_lin_. e) Calculated isolation contrast and peak forward transmission coefficient (|*t*_21_|) for different nonlinear coefficients of the varactor.

Next, we studied the peak isolation contrast, or |*t*_21_|/|*t*_12_|, of the coupled meta‐atom system as a function of input power as well as the specific design parameters of the resonators. When the input power was low, the isolation contrast was 1. The isolation contrast increased as the input power increased for both cases, as shown in Figure 4b. In the case when Δ*ω* = 0.047 and *k* = 0.0031, a higher isolation contrast was achieved, when compared to the case for Δ*ω* = 0 and *k* = 0.001. The maximum isolation contrast achieved at the highest input power was also dependent upon the specific parameters of the resonators. The CMT calculation results agree well with the measured results, thereby providing an approach to investigate the effect of the resonator parameters on the maximum isolation contrast. As shown in Figure 4c, decreasing the coupling factor (*k*) and increasing frequency detuning (Δ*ω*) served to increase the isolation contrast for the highest input power. For a constant *k*, the isolation contrast increased quadratically with increasing frequency detuning. For a constant frequency detuning, the isolation contrast increased linearly with a decrease in the coupling coefficient. This dependence of the isolation contrast on the frequency detuning and coupling coefficient is such that weaker interactions between the two resonators result in a higher isolation contrast. As shown in Figure 4c, the experimental and theoretical results for different configurations of the meta‐atom resonators agree qualitatively, further supporting the validity of the theoretical calculation. Ultimately, given that the forward transmission decreases as the coupling decreases, increasing the isolation contrast necessarily sacrifices the forward transmission coefficient.

The relationship between peak forward transmission and the asymmetry in the coupled resonators may also be quantitatively evaluated. We define *κ*_lin_ as the ratio of the mode amplitude of the nonlinear resonator for excitation from opposing sides when the excitation power is low, that is, in the linear regime, a representation of the asymmetry of the coupled system. The peak forward transmission coefficient is bounded by $t_{21}\leq \frac{2\kappa_{\mathrm{lin}}}{1+\kappa_{\mathrm{lin}}^{2}}$, the derivation of which may be found in the Supporting Information. The estimation agrees well with the general model for asymmetric structures.^[^ ^44^ ^]^ Since the intrinsic loss is neglected in the derivation of the transmission coefficient boundary, the measured transmission coefficients for different configurations of the coupled meta‐atoms fall within the allowed area, as shown in Figure 4d. The asymmetric mode amplitude of the nonlinear resonator for excitation from opposite input ports is the origin of the nonreciprocal response and the larger asymmetric ratio equates to a higher isolation contrast. The bounded transmission coefficient quantitatively describes the aforementioned trade‐off between isolation contrast and the transmission coefficient.

We also assessed the effect of the nonlinear coefficient (*λ*_0_) on the isolation contrast and maximum forward transmission coefficient. In the case of Δ*ω* = 0.047 and *k* = 0.0031, we calculated the isolation contrast and forward transmission coefficient at a fixed incident power of 10 dBm for different nonlinear coefficients by using the CMT model, as shown in Figure 4f. As the nonlinear coefficient increases, the isolation contrast increases as the nonlinearity becomes more pronounced; however, the maximum forward transmission coefficient decreases. Therefore, in addition to the tradeoff between isolation contrast and forward transmission coefficient in selecting the frequency detuning and coupling coefficient detailed above, there is also a requisite tradeoff when selecting varactors with different nonlinear coefficients.

Herein, we developed an analytical model within the framework of the CMT to describe the nonreciprocal response of coupled nonlinear‐linear resonators. We employed magnetically coupled linear and nonlinear helical meta‐atoms to construct the nonreciprocal system. The theoretical model reveals that the nonreciprocal response stems from the asymmetry in the coupled meta‐atoms. Experimental results demonstrate that the nonreciprocity depends on the input power and that the maximum forward–backward isolation contrast approximates 20 dB when the input power is 10 dBm. The CMT‐based model of the nonreciprocal meta‐atoms presented herein has been validated by its high‐degree of agreement with the measured experimental results. In addition, the theoretical model predicted the inverse relationship between the isolation contrast and maximum transmission coefficient and defined the bounds in the transmission coefficient for different orders of asymmetry in the coupled system, all of which were supported by experimentally‐measured results. The analytical model developed in this work is not only valid for nonlinear, nonreciprocal meta‐atoms, but readily generalizable to the design of myriad coupled nonlinear resonating systems, such as radiofrequency integrated circuits, optical metamaterials and metasurfaces, and integrated photonic resonators, among others. As such, the CMT‐based model reported herein may be considered a novel design paradigm in coupled nonlinear resonating systems.

## Experimental Section

##### Theoretical Modeling of The Nonlinear, Nonreciprocal Coupled Meta‐Atoms

In the coupled meta‐atoms, the mode amplitudes *a*_1_ and *a*_2_ were calculated by solving the frequency domain equation (Equation (3)), followed by calculating the forward (*t*_21_) and backward (*t*_12_) transmission coefficients.

In the calculation of the forward transmission, the input signal amplitudes were set as *s*_1+_≠ 0 and *s*_2+_ = 0. From Equation (3), we obtained:

(5) $$\begin{array}{ccc} & & \left\{\left[j\left(\omega -\omega_{1}\right)+\frac{1}{\tau_{e1}}+\frac{1}{\tau_{o1}}\right]\left[j\left(\omega -\omega_{2}\right)+\frac{1}{\tau_{e2}}+\frac{1}{\tau_{o2}}\right]+k^{2}\right\}a_{2}\\ & & \;=jk\sqrt{\frac{2}{\tau_{e1}}}s_{1+} \end{array}$$

In this equation, the resonance frequency *ω*_2_  =  *ω*_*o*2_(1  −  *λ*_0_|*a*_2_|) is linearly dependent on the magnitude of mode amplitude *a*_2,_^[^ ^27^ ^]^ and Equation (5) becomes a nonlinear equation. The nonlinear equation was solved by using the Newton method in MATLAB and the mode amplitude *a*_2_ was obtained. The output signal at port 2 may be calculated by $s_{2-}=\sqrt{2/\tau_{e2}}a_{2}$ and the forward transmission coefficient may be calculated by *t*_21_  =  *s*_2 −_ /*s*_1 +_.

For the backward transmission, the input signal amplitudes were set as *s*_1+_ = 0 and *s*_2+_≠ 0. From Equation (3), we obtained:

(6) $$\begin{array}{ccc} & & \left\{\left[j\left(\omega -\omega_{1}\right)+\frac{1}{\tau_{e1}}+\frac{1}{\tau_{o1}}\right]\left[j\left(\omega -\omega_{2}\right)+\frac{1}{\tau_{e2}}+\frac{1}{\tau_{o2}}\right]+k^{2}\right\}a_{2}\\ & & \;=\left[j\left(\omega -\omega_{1}\right)+\frac{1}{\tau_{e1}}+\frac{1}{\tau_{o1}}\right]\sqrt{\frac{2}{\tau_{e2}}}s_{2+} \end{array}$$

(7) $$a_{1}=\frac{jka_{2}}{j\left(\omega -\omega_{1}\right)+\frac{1}{\tau_{e1}}+\frac{1}{\tau_{o1}}}$$

Equation (6) was numerically solved to obtain the mode amplitude *a*_2_ by using the Newton method and *a*_1_ was calculated from Equation (7). The output signal at port 1 may be calculated as $s_{1-}=\sqrt{2/\tau_{e1}}a_{1}$ and the backward transmission coefficient as *t*_12_  =  *s*_1 −_ /*s*_2 +_ .

The transmission coefficients at different frequencies and input powers were calculated by varying *ω*, *s*_1+_, and *s*_2+_ when solving the equations. The resonance frequencies (*ω*_1_ and *ω*_2_) and the coupling factor (*k*) may also be varied to study the responses for different meta‐atom designs and separation distances. It is to be noted that *τ*_*e*1_ = 228.79 ns, *τ*_*o*1_ = 198.94 ns, *τ*_*e*2_ = 278.52 ns, and *τ*_*o*2_ = 248.68 ns were used in the majority of the calculations in this article if not otherwise specified.

##### Numerical Simulation

To investigate the resonance mode of the coupled nonreciprocal resonators, a finite element model was constructed using CST Studio Suite 2018. The dimensions of the structures in the model mirrored the structures employed in the experiments. In the model, the wire diameter was 0.28 mm, the helical diameter was 50 mm, the pitch in the helix was 1.8 mm for both the linear and nonlinear meta‐atoms, and the number of turns was three in the linear helical meta‐atom for the frequency‐matched case and 2.35 for the nonlinear helical meta‐atoms. In the nonlinear meta‐atom, the varactor‐loaded split‐ring resonator (VLSRR) with a radius of 50 mm was placed 4 mm away from the helical structure. The varactor exhibited a nominal capacitance of 3.2 pF at its original state. Since it was not possible to model the input field‐dependent capacitance of the varactor, the varactor's capacitance was assumed to be 4.5 pF for the high input power in the backward direction in order to model the nonlinear response. The separation distance between the linear and nonlinear meta‐atoms was 250 mm, which was the same as the experimental setup. In the numerical model, the conductivity of copper was set to be 5.97 × 10^5^ S cm^−1^, and the permittivity of the scaffold was 2, with a loss tangent of 0.03.

##### Experimental Characterization

3D printing and copper wire winding were employed to fabricate the helical resonator and form the linear meta‐atoms. The dimensions of the structure were identical to those described in the Numerical Simulation section above. An SMV 2020 varactor (Skyworks Inc.) was soldered into the split‐ring resonator to form a VLSRR, which was assembled into a meta‐atom to yield a nonlinear meta‐atom. According to the data sheet of SMV 2020, the nonlinear coefficient is ≈0.1648. Loop antennas were used as excitation ports in the experiments. We employed a network analyzer (VNA E5071C, Keysight Inc.) to characterize the forward and backward transmission coefficients by measuring the corresponding S‐parameters. The input power was swept from −20 to 10 dBm with a step of 2 dBm to characterize the nonlinear and nonreciprocal response. In the experiments of variations in frequency detuning, the number of turns of the linear resonator was decreased from 3 to 2.25 with a step of 0.25. The separation distance between the linear and nonlinear meta‐atoms was also altered to obtain the results for different coupling coefficients.

## Conflict of Interest

The authors have filed a provisional patent application on the work described herein.

## Supporting information

Supporting Information
