# Supplementary material for: Nonreciprocal Magnetic Coupling Using Nonlinear Meta‐Atoms
Source: Adv Sci (Weinh). 2020 Jul 23;7(19):2001443. doi: 10.1002/advs.202001443 (PMC7539216; doi:10.1002/advs.202001443)
Supplement: Supplementary file 1 — Supporting Information [file ADVS-7-2001443-s001.pdf]

Copyright WILEY-VCH Verlag GmbH & Co. KGaA, 69469 Weinheim, Germany, 2020.

Supporting Information

Nonreciprocal Magnetic Coupling Using Nonlinear Meta-Atoms

Xiaoguang Zhao, Ke Wu, Chunxu Chen, Thomas G. Bifano, Stephan W. Anderson, and Xin Zhang

1. Deriving the bound in peak forward transmission coefficient.

In the nonlinearity-based non-reciprocal coupled meta-atoms, the non-reciprocity stems from the asymmetry in the coupled resonators. We define the ratio of the mode amplitude of the nonlinear resonator for excitation from opposing sides in the linear regime as κ_{lin}. In the linear regime, the system equation in the frequency domain is:

$$j\omega a_1 = \left[j\omega_1 - \frac{1}{\tau_{e1}} - \frac{1}{\tau_{01}} \right] a_1 + jka_2 + \sqrt{\frac{2}{\tau_{e1}}} s_{1+} \tag{S1}$$

$$j\omega a_2 = \left[j\omega_2 - \frac{1}{\tau_{e2}} - \frac{1}{\tau_{02}} \right] a_2 + jka_1 + \sqrt{\frac{2}{\tau_{e2}}} s_{2+} \tag{S2}$$

When the system is excited from port 1 (i.e. $s_{1+} = 1$, $s_{2+} = 0$), the mode amplitude of resonator 2 is:

$$a_{2,1} = \frac{jk\sqrt{\frac{2}{\tau_{e1}}}}{\left[j(\omega-\omega_1) + \frac{1}{\tau_{e1}} + \frac{1}{\tau_{01}} \right] \left[j(\omega-\omega_2) + \frac{1}{\tau_{e2}} + \frac{1}{\tau_{02}} \right] + k^2} \tag{S3}$$

When the system is excited from port 2 (i.e. $s_{1+} = 0$, $s_{2+} = 1$), the mode amplitude of resonator 2 is:

$$a_{2,2} = \frac{\left[j(\omega-\omega_1) + \frac{1}{\tau_{e1}} + \frac{1}{\tau_{01}} \right] \sqrt{\frac{2}{\tau_{e2}}}}{\left[j(\omega-\omega_1) + \frac{1}{\tau_{e1}} + \frac{1}{\tau_{01}} \right] \left[j(\omega-\omega_2) + \frac{1}{\tau_{e2}} + \frac{1}{\tau_{02}} \right] + k^2} \tag{S4}$$

Therefore, we obtain at the resonant mode:

$$\kappa_{lin} = \frac{|a_{2,2}|}{|a_{2,1}|} = \frac{1}{k} \sqrt{\frac{1}{\tau_{e1}\tau_{e2}}} \tag{S5}$$

The forward transmission coefficient (t_{21}) may be calculated by:

$$t_{21} = \frac{jk\sqrt{\frac{2}{\tau_{e1}}}\sqrt{\frac{2}{\tau_{e2}}}}{\left[j(\omega-\omega_1) + \frac{1}{\tau_{e1}} + \frac{1}{\tau_{01}} \right] \left[j(\omega-\omega_2) + \frac{1}{\tau_{e2}} + \frac{1}{\tau_{02}} \right] + k^2} \tag{S6}$$

The maximum forward transmission coefficient is:

$$|t_{21}|_{\max} = \frac{2k\sqrt{\tau_{e1}\tau_{e2}}}{1 + k^2\tau_{e1}\tau_{e2}} \tag{S7}$$

Therefore, we obtain:

$$|t_{21}| \le \frac{2\kappa_{lin}}{1 + \kappa_{lin}^2} \tag{S8}$$

The bound of the transmission coefficient for different degrees of asymmetry is shown in Figure 4d in the main text.
